# Square-Root Unscented Information Filter and Its Application in SINS/DVL Integrated Navigation

**DOI:** 10.3390/s18072069

**Published:** 2018-06-28

**Authors:** Yan Guo, Meiping Wu, Kanghua Tang, Lu Zhang

**Affiliations:** National University of Defense Technology, Deya Road No. 109, Kaifu District, Changsha 410073, China; tt_kanghua@hotmail.com (K.T.); zhanglu_nudter@163.com (L.Z.)

**Keywords:** SINS/DVL integrated navigation, unscented information filter, square root, state probability approximation, most suitable parameter form

## Abstract

To address the problem of low accuracy for the regular filter algorithm in SINS/DVL integrated navigation, a square-root unscented information filter (SR-UIF) is presented in this paper. The proposed method: (1) adopts the state probability approximation instead of the Taylor model linearization in EKF algorithm to improve the accuracy of filtering estimation; (2) selects the most suitable parameter form at each filtering stage to simply the calculation complexity; (3) transforms the square root to ensure the symmetry and positive definiteness of the covariance matrix or information matrix, and then to enhance the stability of the filter. The simulation results indicate that the estimation accuracy of SR-UIF is higher than that of EKF, and similar to UKF; meanwhile the computational complexity of SR-UIF is lower than that of UKF.

## 1. Introduction

Most underwater or surface navigation applications employ a Strapdown Inertial Navigation System (SINS) as their main navigation sensor, since SINS is a standalone system that can provide all of the required navigation data: position, velocity and orientation [1,2,3]. However, even with high precision SINS, the navigation solutions drift in time due to measurement errors of its inertial sensors. The Doppler velocity log (DVL) is a good acoustic-based device in marine applications, which can provide three-dimensional velocities to mitigate the errors of marine SINS [4,5,6]. Therefore, the integrated SINS and DVL navigation system is a common navigation method for underwater or surface navigation during long voyages [7,8,9]. The SINS system error model is nonlinear, and a nonlinear filtering algorithm is generally used for state estimation. The earliest nonlinear filtering method used in engineering was the Extend Kalman Filter (EKF) algorithm [10,11]. Its core idea is to approximate a linear expansion of the current nonlinear state equation (namely a Taylor series expansion, truncating high-order terms, retaining first-order terms) to apply the rules of EKF. However, the EKF algorithm is only applicable to weakly nonlinear systems. The stronger the nonlinearity of the estimated object is, the larger the estimation error will be, and it will even cause filter divergence.

Some scholars have proposed a probabilistic approximation of the nonlinear filtering construction idea [12], that is, using a deterministic sampling method to replace the Taylor series expansion linearization of the system model in EKF algorithm, and to approach the mean and variance of the Gauss state distribution by utilizing deterministic sampling points through a nonlinear system equation transformation propagation. The Unscented Kalman Filter (UKF) algorithm proposed by Uhlman is the first nonlinear algorithm to practice this idea [13,14,15]. It adopts an Unscented Transformation (UT) to obtain 2n + 1 Sigma sampling points with different weights, and uses the abovementioned Sigma sampling points to generate new points after transforming the nonlinear system equation for estimating the mean and the variance of the system states at the next moment. The theoretical deduction proves that the estimation accuracy of UKF algorithm can reach the third-order terms of Taylor series expansion for the nonlinear system [16,17,18].

The information filtering algorithm realizes a state estimation by transforming information parameters (including information matrix and information vector) [19,20]. It is equivalent to a series of Kalman filter algorithms which pass the moment parameters (covariance matrix and state vector) [21,22]. The whole filtering process can be divided into two processes: time updating and measurement updating. The time updating process involves the calculation of marginal probability, and the retrieval of the moment parameter form is relatively simple. The measurement updating process involves conditional probability, and the information parameter form is more effective. Therefore, in order to optimize the performance of the algorithm, the Square-Root Unscented Information Filter (SR-UIF) algorithm is proposed in this paper, which is applied to a nonlinear integrated SINS/DVL navigation system. This algorithm adopts the form of moment parameters in the process of time updating according the characteristic of the parameter form, respectively, the form of information parameters in the process of measurement updating; and ensures the symmetry and positive definite of the information matrix or covariance matrix by propagating their square root, and alleviates the problems such as divergence and data precision degradation caused by rounding errors in the filtering process.

The remainder of this paper is organized as follows: Section 2 studies the duality of Gaussian distribution; Section 3 focuses on the theoretical derivation of the SR-UIF algorithm; Section 4 briefly introduces the SINS/DVL model used in this paper; Section 5 verifies our findings through simulations and the conclusions are given in Section 6.

## 2. Duality of a Gaussian Distribution

We set ***X*** as the state vector of the integrated navigation system, which obeys a Gaussian distribution of multi-dimensional random variables, namely $p(X)∼N(\hat{X},P)$, $\hat{X}$ is its mean value and ***P*** is its variance. Through the expansion of the Gauss distribution index term, it can be obtained that:

(1) $$\begin{array}{ll} p(X) & =N(\hat{X},P)\\ & =\frac{1}{\sqrt{\left|2\pi P\right|}}\exp\left\{-\frac{1}{2}{\left(X-\hat{X}\right)}^{T}P^{-1}\left(X-\hat{X}\right)\right\}\\ & =\frac{e^{-\frac{1}{2}y^{T}Y^{-1}y}}{\sqrt{\left|2\pi Y^{-1}\right|}}\exp\left\{-\frac{1}{2}X^{T}YX+y^{T}X\right\}\\ & =N^{-1}(y,Y) \end{array}$$

where Y is the information matrix, and y is the information vector. It is usually called $\hat{X}$ and ***P*** as the moment parameter form, y and Y as the information parameter form, and their transformation relations are:

(2) $$\{\begin{array}{c} Y=P^{-1}\\ y=Y\hat{X}=P^{-1}\hat{X} \end{array}$$

From Equations (1) and (2), the information parameter form is also an expression of the Gaussian distribution, which is equivalent to the representation of the moment parameter form, and the two forms can be converted to each other. These are collectively referred to as the duality of the Gaussian distribution. The whole filtering process can be divided into time updating and measurement updating:

(1) the time updating process involves the calculation of marginal probability:
  $$\rho (X_{k}/U^{k-1},Z^{k-1})=\int \rho (X_{k},X_{k-1}/U^{k-1},Z^{k-1})dX_{k-1}$$
(2) The measurement updating process involves the calculation of conditional probability:
  $$\rho (X_{k}/U^{k-1},Z^{k})=\frac{\rho (Z_{k}/X_{k})\rho (X_{k}/U^{k-1},Z^{k-1})}{\int \rho (Z_{k}/X_{k})\rho (X_{k}/U^{k-1},Z^{k-1})dX_{k}}$$

Suppose that ***X*** and ***Z*** satisfy the Gauss distribution as follows:

$$\rho (X,Z)=N\left(\left[\begin{array}{c} {\hat{x}}_{X}\\ {\hat{x}}_{Z} \end{array}\right];\left[\begin{array}{cc} P_{X,X} & P_{X,Z}\\ P_{Z,X} & P_{Z,Z} \end{array}\right]\right)=N^{-1}\left(\left[\begin{array}{c} {\hat{y}}_{X}\\ {\hat{y}}_{Z} \end{array}\right];\left[\begin{array}{cc} Y_{X,X} & Y_{X,Z}\\ Y_{Z,X} & Y_{Z,Z} \end{array}\right]\right)$$

The condition probability and marginal probability of the moment parameter form and information parameter are as shown in Table 1 below. It can be seen when using the moment parameters and information parameters to calculate the marginal probability and conditional probability, the two equivalent expressions have completely different calculation characteristics. It is relatively simple to calculate the marginal probability in the moment parameter form. On the contrary, when calculating conditional probability, it is relatively effective to calculate the conditional probability in the information parameter form.

## 3. Square-Root Unscented Information Filter (SR-UIF) Algorithm

Square-Root Unscented Information Filter (SR-UIF) algorithm is a nonlinear filtering algorithm based on probabilistic approximation, which has the same structure as the well known UKF algorithm. It adopts an Unscented Transformation (UT) to obtain 2n + 1 Sigma sampling points with different weights, and uses the abovementioned Sigma sampling points to generate new points after transforming the nonlinear system equation for estimating the mean and the variance of the system states at the next moment. However, compared with the UKF algorithm, the SR-UIF algorithm selects the most suitable expression form at each filtering stage, that is, using the moment parameter form in the time updating, using the information parameter form in the measurement updating, for simplifying the computational complexity. Meanwhile, SR-UIF algorithm ensures the symmetry and positive definiteness of the information matrix or covariance matrix by propagating their square root, for improving the stability of the algorithm.

The discretized integrated navigation system model is:

(3) $$\{\begin{array}{c} X_{k}=f(X_{k-1})+G_{k-1}W_{k-1}\\ Z_{k}=h\left(X_{k}\right)+V_{k} \end{array}$$

The following is analyzed for the implementation steps of SR-UIF algorithm. First, it is assumed that the initial filter estimation state is as follows:

(4) $$\{\begin{array}{c} {\hat{X}}_{0}=E[X_{0}]\\ P_{0}=E[(X_{0}-{\hat{X}}_{0}){\left(X_{0}-{\hat{X}}_{0}\right)}^{T}] \end{array}$$

Combined with the duality of Gaussian distribution, the initial information parameters can be obtained:

(5) $$\{\begin{array}{c} y_{0}=Y_{0}{\hat{X}}_{0}\\ Y_{0}^{-1}=P_{0}={\left\{E[(X_{0}-{\hat{X}}_{0}){\left(X_{0}-{\hat{X}}_{0}\right)}^{T}]\right\}}^{-1} \end{array}$$

### 3.1. Time Updating

The calculating of the edge probabilities involved in the time updating is simpler by using the moment parameter form. Therefore, the mean and variance are applied as the iterative factors in the time updating process of SR-UIF algorithm. The specific process is as follow:

(1) Decompose the covariance matrix ${\hat{P}}_{k-1}$ at k−1 time by using the Cholesky algorithm:
  (6) $${\hat{P}}_{k-1}=S_{k-1}S_{k-1}^{T}$$
  where $S_{k-1}$ is the Cholesky factor of the covariance matrix ${\hat{P}}_{k-1}$, and is the lower triangular matrix.
(2) Calculate the Sigma point sets and their weights according to the Unscented Transformation (UT):
  (7) $$\{\begin{array}{ll} \;\xi_{i}=0,\;W_{i}=W_{i}^{m}=W_{i}^{c}=\frac{\kappa }{n+\kappa } & ,\;i=0\\ \xi_{i}=\sqrt{\frac{n+\kappa }{2}}{\left[1\right]}_{i},\;W_{i}=W_{i}^{m}=W_{i}^{c}=\frac{1}{2\left(n+\kappa \right)} & ,\;i=1,\;2,\;\cdots ,\;2n \end{array}$$
  where *n* is the system dimension; κ is a free parameter; $W_{i}^{m}$ is the weighted value corresponding to each Sigma point, $W_{i}^{c}$ is the weighted value corresponding to variance matrix, and satisfies $\sum W_{i}^{c}=\sum W_{i}^{m}=1$; ${\left[1\right]}_{i}$ indicates the *i* column or line of identity matrix [1]. Further, the state sample points at k time can be obtained as follows:
  (8) $$X_{i,k-1}=S_{k-1}\xi_{i}+{\hat{x}}_{k-1}$$
(3) One-step state prediction augmented sample points $X_{i,k|k-1}^{*}$ can be obtained through a nonlinear transformation:
  (9) $$X_{i,k|k-1}^{*}=F(X_{i,k-1})$$
  Then their mean and covariance matrix at k time are
  (10) $${\hat{x}}_{k|k-1}=\sum_{i=0}^{m}W_{i}^{m}X_{i,k|k-1}^{*}$$
  (11) $$P_{k/k-1}=\sum_{i=0}^{m}W_{i}^{c}X_{i,k|k-1}^{*}X_{i,k|k-1}^{*T}-{\hat{x}}_{k/k-1}{\hat{x}}_{k/k-1}^{T}+Q_{k-1}$$
  where $Q_{k-1}$ is the variance matrix of system noise.

### 3.2. Measurement Updating

As it is relatively simple to calculate the conditional probabilities by utilizing the information parameter form, the information vector and information matrix are applied as the iterative factors in the measurement updating process of SR-UIF algorithm, for achieving the optimal design of the algorithm. According to the theoretically deduced knowledge in the Appendix, it can be seen that the update equation of the information matrix and the information vector at k time:

(12) $$\{\begin{array}{c} {\hat{Y}}_{k}=Y_{k/k-1}+I_{k}\\ {\hat{y}}_{k}={\hat{y}}_{k/k-1}+i_{k} \end{array}$$

where $Y_{k/k-1}$ is the one-step prediction information matrix, and $Y_{k/k-1}=P_{k/k-1}^{-1}$; ${\hat{y}}_{k/k-1}$ is the one-step prediction information vector, and ${\hat{y}}_{k/k-1}=Y_{k/k-1}{\hat{x}}_{k/k-1}$; $I_{k}$ and $i_{k}$ are the contributions of the information matrix and the information parameters, respectively, and:

(13) $$\{\begin{array}{c} I_{k}=\left(Y_{k/k-1}P_{xz,k/k-1}\right)R_{k}^{-1}{\left(Y_{k/k-1}P_{xz,k/k-1}\right)}^{T}\\ i_{k}=\left(Y_{k/k-1}P_{xz,k/k-1}\right)R_{k}^{-1}\left(z_{k}-h\left({\hat{x}}_{k/k-1}\right)+P_{xz,k/k-1}^{T}{\hat{y}}_{k/k-1}\right) \end{array}$$

$R_{k}^{}$ is the variance matrix of measurement noise, $P_{xz,k/k-1}$ is the cross-covariance matrix between x and z.

It can be seen from Equation (13) that the measurement update states require the known $P_{xz,k/k-1}$. Now the specific solution process of $P_{xz,k/k-1}$ is given as follows:

(1) Decompose the covariance matrix $P_{k/k-1}$ by using the Cholesky algorithm again,
  (14) $$P_{k/k-1}=S_{k/k-1}S_{k/k-1}^{T}$$
  where $S_{k/k-1}$ is the Cholesky factor of the covariance matrix $P_{k/k-1}$, and is the lower triangular matrix.
(2) Calculates the one-step prediction state sample points:
  (15) $$X_{i,k|k-1}=S_{k|k-1}\xi_{i}+{\hat{x}}_{k|k-1}$$
(3) One-step measurement prediction augmented sample points can be obtained through nonlinear transformation:
  (16) $$Z_{i,k|k-1}=h\left(X_{i,k|k-1}\right)$$
  then their mean at k time is:
  (17) $${\hat{z}}_{k|k-1}=\sum_{i=0}^{m}W_{i}^{m}Z_{i,k|k-1}$$
  and the cross-covariance matrix is:
  (18) $$P_{xz,k/k-1}=\sum_{i=0}^{m}W_{i}^{c}X_{i,k|k-1}^{*}Z_{i,k|k-1}^{*T}-{\hat{x}}_{k/k-1}{\hat{z}}_{k/k-1}^{T}$$

In order to facilitate the experiment recording and observation, the filtering finally needs to transform the information parameter form into the moment parameter form. However, the state quantities involved in the inversion of large information matrix are undoubtedly huge, which makes the algorithm difficult to process. For this, this paper utilizes the Cholesky decomposition to deal with it, that is, firstly:

(19) $${\hat{Y}}_{k}=L_{k}L_{k}^{T}$$

where $L_{k}$ is the Cholesky factor of the information matrix ${\hat{Y}}_{k}$.

Since the Cholesky factor $L_{k}$ is a lower triangular matrix and its upper half elements are all zero, the computational complexity of the inverse operation of $L_{k}$ is much less than that of ${\hat{Y}}_{k}$ when both $L_{k}$ and ${\hat{Y}}_{k}$ are the same dimension. It can realize the optimization design of the algorithm for using Cholesky factor $L_{k}$ to solve the state vector estimate at k time:

(20) $${\hat{x}}_{k}={\left(L_{k}^{T}\right)}^{-1}f$$

where f is the forward vector of Cholesky, and $f=L_{k}^{-1}{\hat{y}}_{k}$.

Equation (6) shows that the covariance matrix ${\hat{P}}_{k}$ needs to be decomposed during the next time updating process, and Equation (19) has been decomposed to the information matrix ${\hat{Y}}_{k}$ at the next moment, which is a duplicate calculation. In view of this, the paper applies Equation (19) instead of Equation (6) to achieve further reduce the amount of the filtering calculation according to the transformation relation between ${\hat{P}}_{k}$ and ${\hat{Y}}_{k}$. The specific operation is as follows:

(1) According to the transformation relation between ${\hat{P}}_{k}$ and ${\hat{Y}}_{k}$, the equation relationship between $S_{k}$ and $L_{k}$ is:
  (21) $$\{\begin{array}{c} L_{k}={\left(S_{k}^{T}\right)}^{-1}\\ L_{k}^{T}=S_{k}^{-1} \end{array}$$
(2) Then $L_{k}$ is used to instead of $S_{k}$ in Equation (7) for iteration:
  (22) $$X_{i,k}={\left(L_{k}^{T}\right)}^{-1}\xi_{i}+{\hat{x}}_{k}$$

### 3.3. Summary

**Figure 1:**
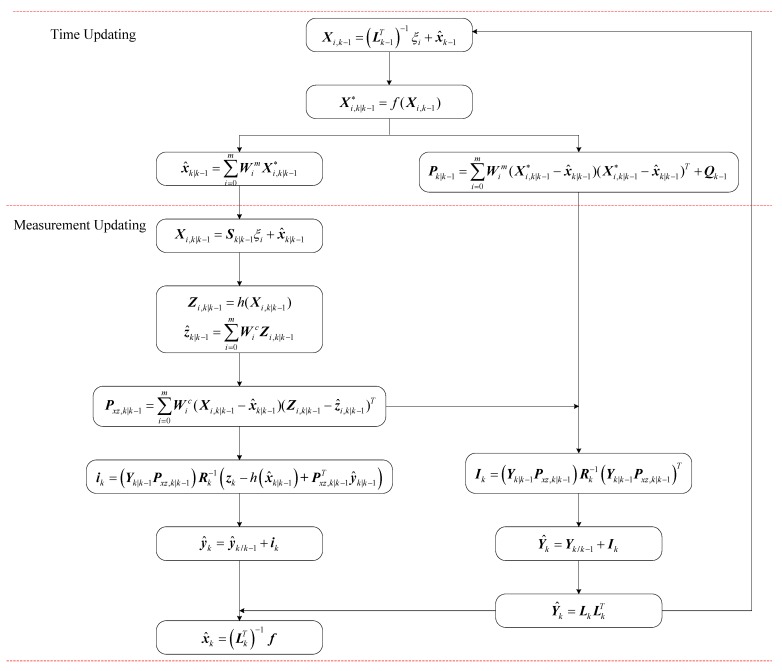
Frame diagram of SR-UIF algorithm.

Based on the above analysis, the frame diagram of SR-UIF algorithm is shown in the following Figure 1.

## 4. Nonlinear SINS/DVL Integrated Navigation Model

Set the local geographic coordinate frame *t* as the navigation frame of SINS, the error propagation equation about misalignment angles $\varphi^{t}$ are constructed as:

(23) $${\dot{\varphi }}^{t}=\delta \omega_{ie}^{t}+\delta \omega_{et}^{t}-\left(\omega_{ie}^{t}+\omega_{et}^{t}\right)\times \varphi^{t}+C_{b}^{t}\varepsilon^{b}$$

where *e*, *b*, and *i* denote the Earth, body, and inertial frames, respectively; $C_{b}^{t}$ is the attitude transformation matrix from the body coordinate frame b to frame *t*; $\varepsilon^{b}$ is constant gyro drift in body frame; $\omega_{ie}^{t}$ is the earth rotational angular rate in frame *t*, and $\delta \omega_{ie}^{t}$ is its calculation error; $\omega_{et}^{t}$ is the rotational angular rate from frame *t* to frame *e* in frame *t*, and $\delta \omega_{et}^{t}$ is it calculation error.

Meanwhile, the vector expression of velocity equation is:

(24) $$\delta {\dot{v}}^{t}=f^{t}\times \varphi^{t}-\left(2\omega_{ie}^{t}+\omega_{et}^{t}\right)\times \delta v^{t}-\left(2\delta \omega_{ie}^{t}+\delta \omega_{et}^{t}\right)\times v^{t}+C_{b}^{t}\nabla^{b}$$

where $\delta v^{t}$ is the velocity error vector; $f^{t}$ is the specific force measured by the accelerometer in frame *t*; $\nabla^{b}$ is the errors of accelerometers in body frame.

Ignoring the height channel, the equations describing position errors are two equations as follow:

(25) $$\left[\begin{array}{c} \delta \dot{L}\\ \delta \dot{\lambda } \end{array}\right]=\left[\begin{array}{c} \frac{\delta v_{y}}{R+h}\\ \frac{\delta v_{x}}{R+h}\sec L+\frac{\delta v_{x}}{R+h}\sec L\tan L\delta L \end{array}\right]$$

where δL and δλ are the latitude and longitude error, respectively.

In this paper, a four-beam phased array DVL is used. The measurement error includes the velocity offset error $\delta V_{d}$, drift angle error δΔ, and calibration coefficient error δC. Assuming that δC is a random constant, $\delta V_{d}$ and δΔ are represented by the first-order Markov process, the error equations of DVL are expressed as:

(26) $$\{\begin{array}{c} \delta {\dot{V}}_{d}=-\beta_{d}\delta V_{d}+\omega_{d}\\ \delta \dot{\Delta }=-\beta_{\Delta }\delta \Delta +\omega_{\Delta }\\ \delta \dot{C}=0 \end{array}$$

where $\beta_{d}^{-1}$, $\beta_{\Lambda }^{-1}$ are expressed as the correlation time of DVL velocity offset error and bias angle error, respectively; $\omega_{d}$ and $\omega_{\Delta }$ are their Gauss white noise.

The system vector is defined as $X=\left[{\begin{array}{cccccccccc} \delta L & \delta \lambda & \delta v_{e} & \delta v_{n} & \varphi_{e} & \varphi_{n} & \varphi_{u} & \delta V_{d} & \delta \Delta & \delta C \end{array}}^{T}\right]$, and the model of the system state equation is applied in this paper:

(27) $$\dot{X}=f(X)+GW$$

where $W={\left[\begin{array}{cccccccccc} 0 & 0 & \omega_{ae} & \omega_{an} & 0 & 0 & 0 & \omega_{d} & \omega_{\Delta } & 0 \end{array}\right]}^{T}$ is the process noise sequence; the specific expressions of and refer to Equations (23)–(26).

Assuming that the DVL measurement is the ground velocity $V_{d}^{\prime }$, then the components of $V_{d}^{\prime }$ in the east and north directions are:

(28) $$\{\begin{array}{c} V_{de}^{\prime }=\left(1+\delta C\right)\left(V_{d}+\delta V_{d}\right)\sin\left(K_{d}+\phi_{u}+\delta \Delta \right)\\ V_{dn}^{\prime }=\left(1+\delta C\right)\left(V_{d}+\delta V_{d}\right)\cos\left(K_{d}+\phi_{u}+\delta \Delta \right) \end{array}$$

where $\phi_{u}$ is the azimuth misalignment angle; $K_{d}$ is the heading angle added to the drift angle. The Taylor series expansion is performed on $x=K_{d}$ for Equation (28):

(29) $$\{\begin{array}{c} V_{de}^{\prime }\approx V_{e}+V_{n}\left(\phi_{u}+\delta \Delta \right)+\delta V_{d}\sin K_{d}+\delta CV_{e}\\ V_{dn}^{\prime }\approx V_{n}-V_{e}\left(\phi_{u}+\delta \Delta \right)+\delta V_{d}\cos K_{d}+\delta CV_{n} \end{array}$$

Meanwhile, the computing velocity of SINS can be expressed as:

(30) $$\{\begin{array}{c} V_{se}^{}=V_{e}+\delta V_{e}\\ V_{sn}^{}=V_{n}+\delta V_{n} \end{array}$$

The difference between the SINS computing velocity and the DVL measurement velocity is taken as the measurement vectors, namely

(31) $$\{\begin{array}{c} \delta V_{e}^{}=\delta V_{e}-V_{n}\left(\phi_{u}+\delta \Delta \right)-\delta V_{d}\sin K_{d}-\delta CV_{e}\\ \delta V_{n}^{}=\delta V_{n}+V_{e}\left(\phi_{u}+\delta \Delta \right)-\delta V_{d}\cos K_{d}-\delta CV_{n} \end{array}$$

Then the measurement equation is set up as follows:

(32) Z=HX+V

where the measurement noise is taken as $V={\left[\begin{array}{cc} v_{e} & v_{n} \end{array}\right]}^{T}$; H is the measurement matrix, and

$$H=\left[\begin{array}{cccccccccc} 0 & 0 & 1 & 0 & 0 & 0 & -V_{n} & -\sin K_{d} & -V_{n} & -V_{e}\\ 0 & 0 & 0 & 1 & 0 & 0 & V_{e} & -\cos K_{d} & V_{e} & -V_{n} \end{array}\right]$$

## 5. Results

In the simulation experiments, two classical motion models are designed: uniform linear motion and uniform circular motion. First of all, the accuracy of position and velocity for purely inertial navigation, filtering with the SR-UIF algorithm, UKF algorithm, and EKF algorithm are compared and analyzed in these two modes of motion. Then, the computational complexity of SR-UIF algorithm and UKF algorithm are also compared and analyzed, which is determined by the average time consumed by a once filtering operation and the total elapsed time of each simulation experiment. Finally, the filtering performance of 1 h off-line data is used to further analyze the performance advantages of SR-UIF algorithm in terms of estimation accuracy and computational complexity. Finally, the filtering performance of 1 h off-line data is used to further analyze the performance advantages of SR-UIF algorithm in terms of estimation accuracy and computational complexity.

### 5.1. Simulation Analysis

Set the simulation conditions: initial latitude $\varphi_{0}=45.7796^{\circ }$, and initial longitude $\lambda_{0}=126.6705^{\circ }$; initial position error δL=δλ=100/R rad, initial velocity error 0.01 m/s; initial misalignment angles $\phi_{e0}=\phi_{n0}=\phi_{u0}=1^{\circ }$; the gyro constant drifts along three axes of body frame are $0.01^{\circ }/h$ with white noise $0.005^{\circ }/h$; the accelerometer biases along three axes of body frame are $1\times 10^{-4}\;g$ with white noise $0.5\times 10^{-4}\;g$; for DVL, the velocity offset error $\delta V_{d}=0.01\;m/s$, the drift angle error $\delta \Delta =1^{\prime }$, the calibration coefficient error δC=0.001, the correlation time of DVL velocity offset error and bias angle error $\beta_{d}^{-1}=5\;\min$, $\beta_{\Delta }^{-1}=15\;\min$. The measurement data are obtained from IMU at a rate of 100 Hz and from DVL at a rate of 1 Hz. The filtering period is 1 s and the simulation time is 6284 s.

The initial parameters are set as:

$${\hat{X}}_{0}={\left[\begin{array}{cccccccccc} 0 & 0 & 0 & 0 & 0 & 0 & 0 & 0 & 0 & 0 \end{array}\right]}^{T}$$

$$P_{0}=diag\left\{\begin{array}{cccccccccc} {\left(100/R\right)}^{2} & {\left(100/R\right)}^{2} & {\left(0.1\;m/s\right)}^{2} & {\left(0.1\;m/s\right)}^{2} & {\left(1^{\circ }\right)}^{2} & {\left(1^{\circ }\right)}^{2} & {\left(1^{\circ }\right)}^{2} & {\left(0.005\;m/s\right)}^{2} & {\left(1^{\prime }\right)}^{2} & {\left(0.001\right)}^{2} \end{array}\right\}$$

$$Q_{0}=diag\left\{\begin{array}{cccccccccc} 0 & 0 & {\left(50\;\mathrm{ug}\right)}^{2} & {\left(50\;\mathrm{ug}\right)}^{2} & {\left(0.005^{\circ }/h\right)}^{2} & {\left(0.005^{\circ }/h\right)}^{2} & {\left(0.005^{\circ }/h\right)}^{2} & q\delta V_{d} & q\delta \Delta & 0 \end{array}\right\}$$

$$R_{0}^{}=diag\left\{\begin{array}{cc} {\left(0.01\;m/s\right)}^{2} & {\left(0.01\;m/s\right)}^{2} \end{array}\right\}$$

where $q\delta V_{d}={\left(0.005\;m/s\right)}^{2}\left(1-e^{-2\beta_{d}T}\right)$, $q\delta \Delta ={\left(1^{\prime }\right)}^{2}\left(1-e^{-2\beta_{\Delta }T}\right)$.

#### 5.1.1. Uniform Linear Motion

**Figure 2:**
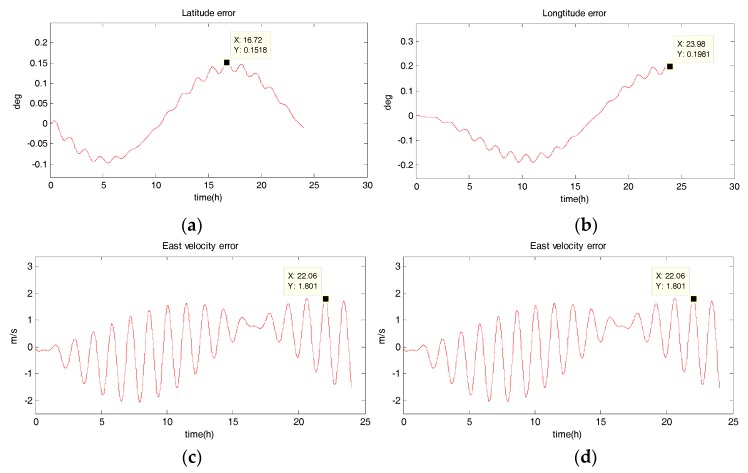
Estimation error curves for purely SINS, where X axis represents simulation time, and Y axis represents: (**a**) latitude error; (**b**) longitude error; (**c**) east velocity error; (**d**) north velocity error.

**Figure 3:**
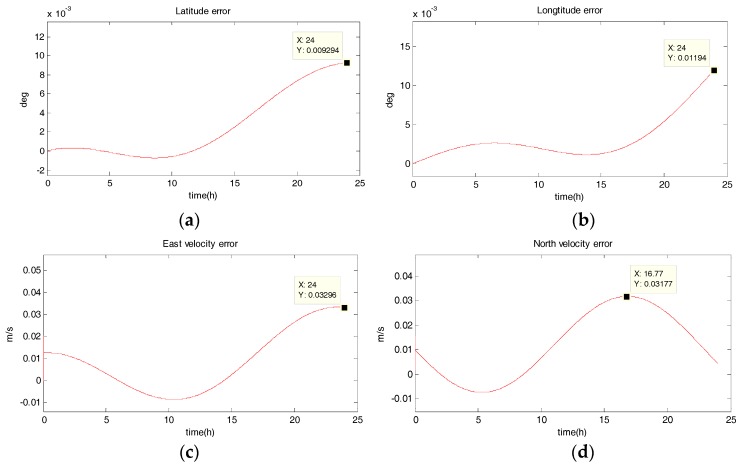
Estimation error curves after EKF, where X axis represents simulation time, and Y axis represents: (**a**) latitude error; (**b**) longitude error; (**c**) east velocity error; (**d**) north velocity error.

**Figure 4:**
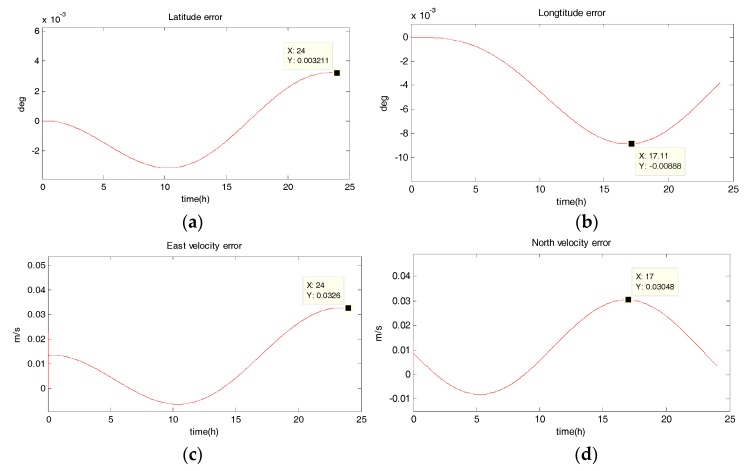
Estimation error curves after UKF, where X axis represents simulation time, and Y axis represents: (**a**) latitude error; (**b**) longitude error; (**c**) east velocity error; (**d**) north velocity error.

**Figure 5:**
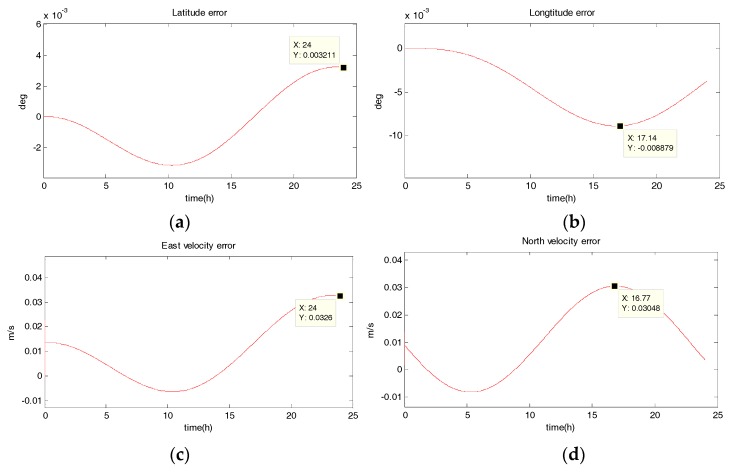
Estimation error curves after SR-UIF, where X axis represents simulation time, and Y axis represents: (**a**) latitude error; (**b**) longitude error; (**c**) east velocity error; (**d**) north velocity error.

The system is in uniform linear motion at 10 m/s with an initial heading angle of 45°, and the simulation time is 24 h. Figure 2, Figure 3, Figure 4 and Figure 5 show the position and velocity estimation error curves for purely inertial navigation, filtering with EKF, UKF, SR-UIF. Table 2 gives the estimation error values.

From the above simulation results in the uniform linear motion, it can be seen that:

(1) The position and velocity errors for purely inertial navigation output diverge with time. The maximum latitude error and longitude error after 24 h are 0.1518° and 0.1981°, meaning 16,888 m and 23,000 m. Meanwhile, the maximum east velocity error and north velocity error reach 1.801 m/s and −2.517 m/s, respectively.
(2) The position estimation errors in SINS/DVL integrated navigation are suppressed after filtering. The maximum latitude error and longitude error after EKF filtering are 9.294 × 10^−30^ and 1.194 × 10^−20^, meaning 720 m and 1322 m. The position estimation accuracy of UKF and SR-UIF algorithm is approximately the same, both higher than that of EKF algorithm. Specifically, the maximum latitude error and longitude error after SR-UIF filtering are 3.211 × 10^−30^ and 8.879 × 10^−20^, meaning 249 m and 986 m.
(3) The velocity estimation errors in SINS/DVL integrated navigation are also suppressed after filtering. The maximum velocity error can be maintained in 10^−2^ order of magnitude, and the velocity estimation accuracy of UKF or SR-UIF algorithm is slightly higher than that of EKF.

#### 5.1.2. Uniform Circular Motion

**Figure 6:**
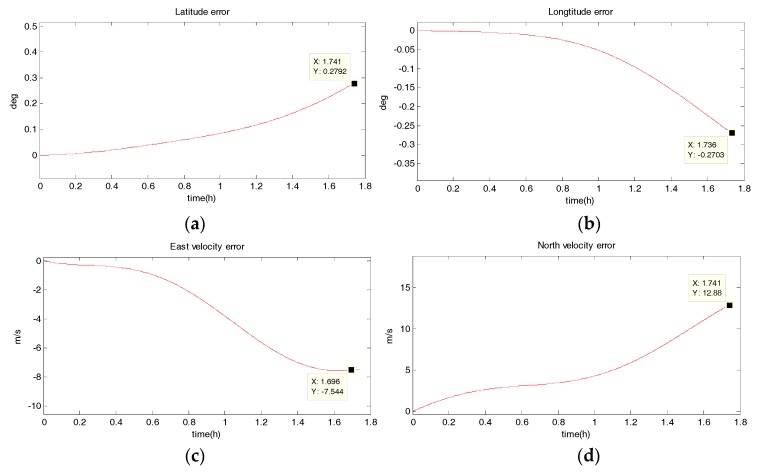
Estimation error curves for purely SINS, where X axis represents simulation time, and Y axis represents: (**a**) latitude error; (**b**) longitude error; (**c**) east velocity error; (**d**) north velocity error.

**Figure 7:**
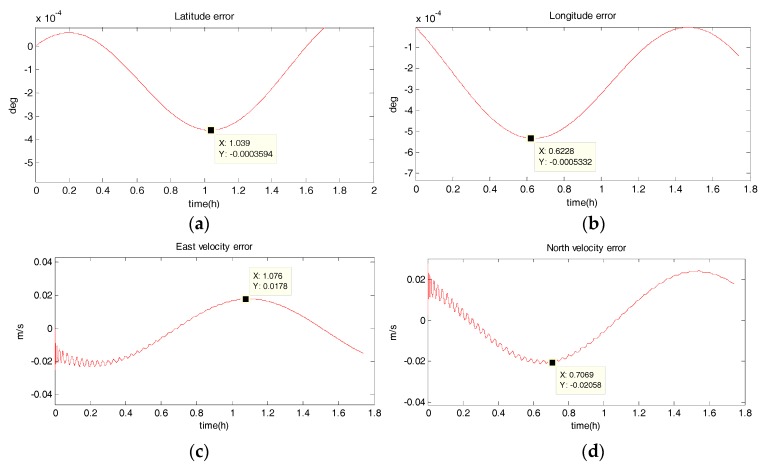
Estimation error curves after EKF, where X axis represents simulation time, and Y axis represents: (**a**) latitude error; (**b**) longitude error; (**c**) east velocity error; (**d**) north velocity error.

**Figure 8:**
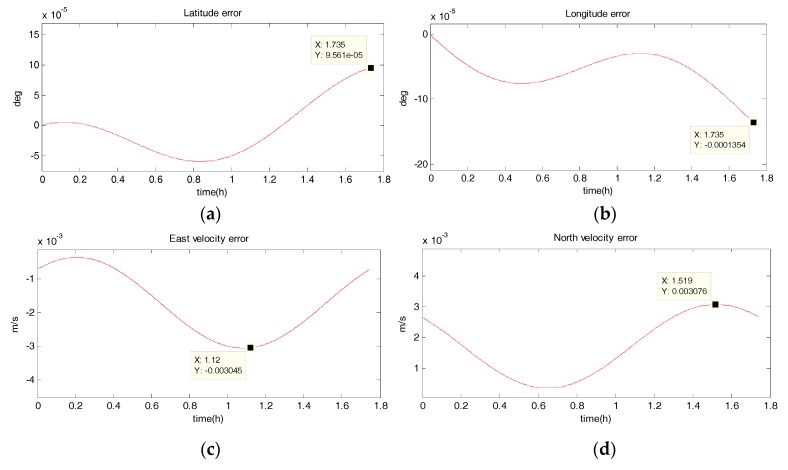
Estimation error curves for purely SINS, where X axis represents simulation time, and Y axis represents: (**a**) latitude error; (**b**) longitude error; (**c**) east velocity error; (**d**) north velocity error.

**Figure 9:**
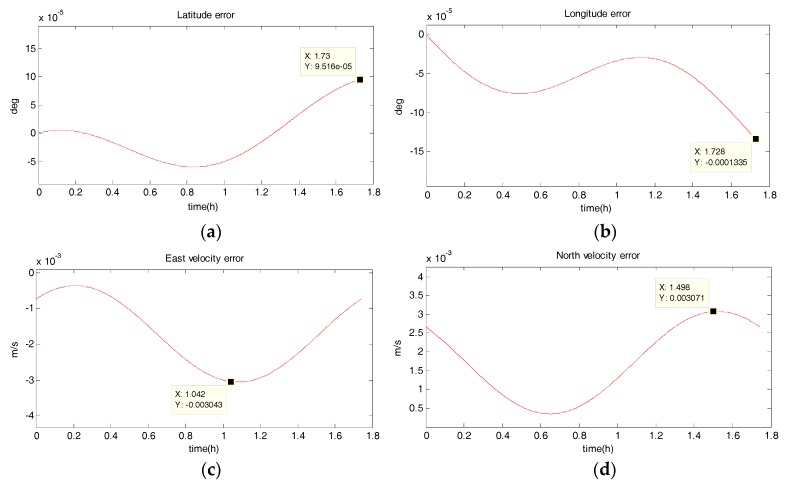
Estimation error curves after EKF, where X axis represents simulation time, and Y axis represents: (**a**) latitude error; (**b**) longitude error; (**c**) east velocity error; (**d**) north velocity error.

The system has a radius of 10 km, a velocity of 10 m/s, an angular rate of $\omega =10^{-3}\;\mathrm{rad}/s$, and performs a uniform circular motion counterclockwise. The simulation results are shown in the following figure. Figure 6, Figure 7, Figure 8 and Figure 9 show the position and velocity estimation error curves for purely inertial navigation, filtering with EKF, UKF, SR-UIF. Table 3 gives the estimation error values.

From the above simulation results, it can be seen that:

(1) Compared to the uniform linear motion, the uniform circular motion is more complex. Then the accuracy of pure inertial navigation is lower in the uniform circular motion for a short time, of which the maximum latitude error and longitude error are 0.2792° and −0.2684°, meaning 31,056 m and 30,302 m; and the maximum east velocity error and north velocity error reach −7.477 m/s and 12.88 m/s, respectively.
(2) The position accuracy is obviously improved after filtering, the maximum latitude error and longitude error after EKF filtering are −3.594 × 10^−40^ and −5.332 × 10^−40^, meaning 28 m and 59 m. The position estimation accuracy of UKF and SR-UIF algorithm is approximately the same, which can be controlled within the same order of magnitude and both higher than that of EKF algorithm.
(3) The velocity error can converge to a small range. The maximum velocity error after EKF filtering can be maintained in 10^−2^ order of magnitude, and the initial filtering stage has obvious oscillatory process. The velocity estimation accuracy of UKF and SR-UIF algorithm advance by an order of magnitude compared to that of EKF, which can be maintained in 10^−3^ order of magnitude and the entire filtering process is smooth.

#### 5.1.3. Performance Analysis

**Figure 10:**
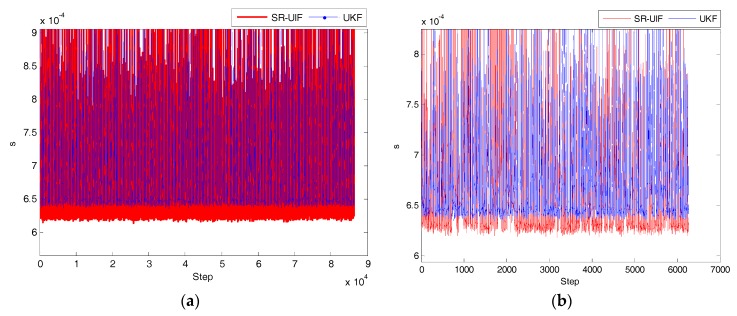
Time comparison consumed by once filtering operation with SR-UIF and UKF: (**a**) in uniform linear motion; (**b**) in uniform circular motion, where X axis represents simulation iterative step, and Y axis represents the time consumed by once filtering operation.

In order to analyze the superiority of SR-UIF algorithm performance, the simulation effect diagram of the time consumed by once filtering operation with SR-UIF and UKF in uniform linear motion or uniform circular motion is given in Figure 10.

We simulate multiple uniform linear motion and uniform circular motion experiments to calculate the average time consumed by once filtering operation with SR-UIF and UKF, and the total elapsed time of each simulation experiment. The results are shown in the following Table 4.

From the above simulation results, it can be seen that whether the system is in a uniform linear motion or a uniform circular motion, the average time consumed by once filtering operation and the total elapsed time of each simulation experiment with SR-UIF algorithm are slightly smaller than with the UKF algorithm, which indicates that in view of the face that SR-UIF algorithm adopts the most suitable parameter form in each filtering stage, the computation amount of filtering is less than that of UKF algorithm.

### 5.2. Measured Data Analysis

**Figure 11:**
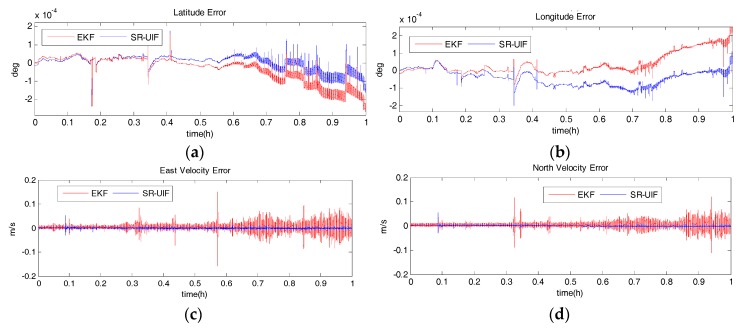
Error curves of SINS/DVL integrated navigation system with SR-UIF and EKF, where X axis represents simulation time, and Y axis represents: (**a**) latitude error; (**b**) longitude error; (**c**) east velocity error; (**d**) north velocity error.

Based on a ship-borne experiment using laboratory fiber optic gyro (FOG) strapdown inertial navigation equipment and a RDI Workhorse-type DVL instrument, the accuracy of the SR-UIF algorithm is analyzed. Figure 11 shows the velocity and position error curves of SINS/DVL integrated navigation system using SR-UIF algorithm and EKF algorithm. It can be seen that in the one-hour data analysis process, the latitude error and longitude error only reach −1.5 × 10^−40^ and −1 × 10^−40^ with SR-UIF algorithm, while these errors reach −2.8 × 10^−40^ and −2.3 × 10^−40^ with EKF algorithm. Meanwhile, the velocity error with SR-UIF algorithm has converged to within 0.02 m/sand remains stable, while within 0.1 m/s with EKF algorithm. The above results show that the positioning accuracy and velocity accuracy of SR-UIF algorithm are higher than that of EKF algorithm.

The estimation accuracy and the time consumed for data analysis of SR-UIF algorithm and UKF algorithm are given below in different data collection time, as shown in Table 5, Table 6 and Table 7.

As can be seen from the data in Table 5, Table 6 and Table 7, the positioning accuracy and velocity accuracy of the SR-UIF algorithm are nearly identical as with the UKF algorithm, but for the total elapsed time in the same collection time, the SR-UIF algorithm is significantly lower than the UKF algorithm.

### 5.3. Discussion

The simulation results and the measured data analysis results show that:

(1) The position and velocity estimation accuracy of the nonlinear filtering based on the probabilistic approximation (such as UKF algorithm and SR-UIF algorithm) is higher than that of EKF algorithm based on the model Taylor series expansion.
(2) SR-UIF algorithm and UKF algorithm are different expressions of the same filtering algorithm based on the probabilistic approximation, and they are equivalent in the filtering estimation value due to the duality of Gaussian distribution. The difference is that SR-UIF algorithm adopts the most suitable parameter form in each filtering stage, making its computational complexity lower than that of UKF algorithm. The simulation results are reflected that the average time consumed by once filtering operation and the total elapsed time of each experiment with SR-UIF algorithm are slightly smaller than UKF algorithm.

## 6. Conclusions and Future Works

In this paper, a square-root unscented information filter (SR-UIF) algorithm is applied to a SINS/DVL integrated navigation system. It is shown that: (1) the algorithm based on probability approximation has a relatively high estimation accuracy, and (2) the performance advantage of the algorithm is optimized by utilizing the most suitable parameter form in each filtering stage, and 3) using the square root of the variance matrix as the iterative factor to ensure the symmetry and positive definite of the information matrix or covariance matrix and thereby enhance the stability of the filtering. Finally, the simulation experiments show that: (1) the positioning and velocity measurement accuracy of SR-UIF algorithm are obviously higher than that of EKF algorithm, equaling to that of UKF algorithm; (2) and the computational complexity of SR-UIF algorithm is slightly lower than that of UKF algorithm. The simulation results provide a good theoretical basis and solution for popularizing the application of SINS/DVL integrated navigation filtering algorithm.

What we touched on in this paper is just the beginning, and there are many places where SR-UIF algorithm can wait for mining applications. The SR-UIF algorithm adopts the form of moment parameters in the process of time updating and the form of information parameters in the process of measurement updating, which makes it convenient to realize distributed or decentralized design of measurement data structure. The potential benefits of this data structure are: (1) improving the fault tolerance of the algorithm by detecting the accuracy of data measured from different sensors; (2) fusing the time unsynchronized measurement information by processing the distributed data. At the same time, the application scene of SR-UIF algorithm can also be transferred from underwater navigation of SINS/DVL to the cooperative navigation system on the ground or in the air, which will also become a direction of the future SR-UIF extension application.

## Figures and Tables

**Table 1:** Expressions for the condition probability and marginal probability of the different form.

Parameter Form	Marginal Probability	Conditional Probability
Moment parameter	$\begin{array}{l} \hat{x}={\hat{x}}_{X}\\ P=P_{X,X} \end{array}$	$\begin{array}{l} \hat{x}={\hat{x}}_{X}+P_{X,Z}P_{Z,Z}^{-1}\left(Z-{\hat{x}}_{Z}\right)\\ P=P_{X,X}-P_{X,Z}P_{Z,Z}^{-1}P_{Z,X} \end{array}$
Information parameter	$\begin{array}{l} \hat{y}={\hat{y}}_{X}-Y_{X,Z}Y_{Z,Z}^{-1}{\hat{y}}_{Z}\\ Y=Y_{X,X}-Y_{X,Z}Y_{Z,Z}^{-1}Y_{Z,X} \end{array}$	$\begin{array}{l} \hat{y}={\hat{y}}_{X}-Y_{X,Z}Z\\ P=Y_{X,X} \end{array}$

**Table 2:** Comparison of position and velocity errors for purely inertial navigation, filtering with EKF, UKF, SR-UIF in uniform linear motion.

Filtering Method	Maximum Latitude Error (deg)	Maximum Longitude Error (deg)	Maximum East Velocity Error (m/s)	Maximum North Velocity Error (m/s)
purely SINS	0.1518	0.1981	1.801	−2.517
EKF	9.294 ×10^−3^	1.194 × 10^−2^	0.03296	0.03177
UKF	3.211 × 10^−3^	8.888 × 10^−3^	0.0326	0.03048
SR-UIF	3.211 × 10^−3^	8.879 × 10^−3^	0.0326	0.03048

**Table 3:** Comparison of position and velocity errors for purely inertial navigation, filtering with EKF, UKF, SR-UIF in uniform circular motion.

Filtering Method	Maximum Latitude Error (deg)	Maximum Longitude Error (deg)	Maximum East Velocity Error (m/s)	Maximum North Velocity Error (m/s)
purely SINS	0.2792	−0.2703	−7.544	12.88
EKF	−3.594 × 10^−4^	−5.332 × 10^−4^	0.0178	0.2058
UKF	9.561 × 10^−5^	1.3254 × 10^−5^	−0.003045	0.003076
SR-UIF	9.561 × 10^−5^	1.3235 × 10^−5^	−0.003043	0.003071

**Table 4:** Comparison of the average time consumed by once filtering operation, and the total elapsed time of each simulation experiment with UKF, SR-UIF in uniform linear motion or uniform circular motion.

Filtering Method	Uniform Linear Motion (24 h)		Uniform Circular Motion (1.67 h)	
	Average Time Consumed by Once Filtering Operation (s)	Total Elapsed Time of Each Simulation Experiment (s)	Average Time Consumed by Once Filtering Operation (s)	Total Elapsed Time of Each Simulation Experiment (s)
UKF	6.413 × 10^−4^	55.40832	6.396 × 10^−4^	4.0083732
SR-UIF	6.212 × 10^−4^	53.67168	6.195 × 10^−4^	3.8824065

**Table 5:** Performance comparison of SR-UIF and UKF in one hour collection data.

Filtering Method	Maximum Latitude Error (deg)	Maximum Longitude Error (deg)	Maximum East Velocity Error (m/s)	Maximum North Velocity Error (m/s)	Total Elapsed Time of Data Analysis (s)
UKF	−2.707 × 10^−5^	1.081 × 10^−4^	−0.02059	0.05312	1.841076
SR-UIF	−2.693 × 10^−4^	9.264 × 10^−5^	−0.02052	0.05304	1.834968

**Table 6:** Performance comparison of SR-UIF algorithm and UKF algorithm in one three collection data.

Filtering Method	Maximum Latitude Error (deg)	Maximum Longitude Error (deg)	Maximum East Velocity Error (m/s)	Maximum North Velocity Error (m/s)	Total Elapsed Time of Data Analysis (s)
UKF	−1.055 × 10^−3^	1.103 × 10^−3^	0.02574	0.05312	3.294395
SR-UIF	−9.494 × 10^−4^	1.0843 × 10^−3^	0.02569	0.05304	3.267851

**Table 7:** Performance comparison of SR-UIF algorithm and UKF algorithm in one five collection data.

Filtering Method	Maximum Latitude Error (deg)	Maximum Longitude Error (deg)	Maximum East Velocity Error (m/s)	Maximum North Velocity Error (m/s)	Total Elapsed Time of Data Analysis (s)
UKF	−1.888 × 10^−3^	1.501 × 10^−3^	−0.03409	0.05312	4.7329875
SR-UIF	−1.795 × 10^−3^	1.446 × 10^−3^	−0.03272	0.05304	4.7236778

## Appendix A

A. Verify that the update variance of the information matrix at k+1 time is:

(A1) $${\hat{Y}}_{k}=Y_{k/k-1}+I_{k}$$

where $I_{k}=\left(Y_{k/k-1}P_{xz,k/k-1}\right)R_{k-1}^{-1}{\left(Y_{k/k-1}P_{xz,k/k-1}\right)}^{T}$.

**Proof.** The approximate estimate form of the covariance matrix $P_{k}$ based on the linear minimum variance is:

(A2) $$P_{k}=P_{k/k-1}-P_{xz,k/k-1}P_{zz,k/k-1}^{-1}P_{xz,k/k-1}^{T}$$

Perform inverse calculations on both sides of Equation (A2),

$$P_{k}^{-1}={\left(P_{k/k-1}-P_{xz,k/k-1}P_{zz,k/k-1}^{-1}P_{xz,k/k-1}^{T}\right)}^{-1}$$

According to the matrix inversion formula, it can be obtained:

(A3) $$\begin{array}{ll} P_{k}^{-1} & ={\left(P_{k/k-1}-P_{xz,k/k-1}P_{zz,k/k-1}^{-1}P_{xz,k/k-1}^{T}\right)}^{-1}\\ & =P_{k/k-1}^{-1}+P_{k/k-1}^{-1}P_{xz,k/k-1}\left(P_{zz,k/k-1}-P_{xz,k/k-1}^{T}P_{k/k-1}^{-1}P_{xz,k/k-1}\right)P_{xz,k/k-1}^{T}P_{k/k-1}^{-1} \end{array}$$

Simultaneously it exits:

$$\begin{array}{l} P_{xz,k/k-1}=\sum_{i=0}^{m}W_{i}^{c}X_{i,k|k-1}^{*}Z_{i,k|k-1}^{*T}-{\hat{x}}_{k/k-1}{\hat{z}}_{k/k-1}^{T}\\ P_{k/k-1}=\sum_{i=0}^{m}W_{i}^{c}X_{i,k|k-1}^{*}X_{i,k|k-1}^{*T}-{\hat{x}}_{k/k-1}{\hat{x}}_{k/k-1}^{T}+Q_{k-1} \end{array}$$

Since X, Z and W are not related to each other, then

$$\begin{array}{l} P_{xz,k/k-1}^{T}P_{k/k-1}^{-1}P_{xz,k/k-1}\\ =\left(\sum_{i=1}^{m}W_{i}^{c}Z_{i,k/k-1}^{*}X_{i,k/k-1}^{*T}-{\hat{z}}_{k/k-1}{\hat{x}}_{k/k-1}^{T}\right)\cdot {\left(\sum_{i=1}^{m}W_{i}^{c}X_{i,k/k-1}^{*}X_{i,k/k-1}^{*T}-{\hat{x}}_{k/k-1}{\hat{x}}_{k/k-1}^{T}+Q_{k-1}\right)}^{-1}\cdot \left(\sum_{i=1}^{m}W_{i}^{c}X_{i,k/k-1}^{*}Z_{i,k/k-1}^{*T}-{\hat{x}}_{k/k-1}{\hat{z}}_{k/k-1}^{T}\right)\\ =\left[W_{1}^{c}Z_{1,k/k-1}^{*}X_{i,k/k-1}^{*T}{\left(X_{1,k/k-1}^{*T}\right)}^{-1}{\left(X_{1,k/k-1}^{*}\right)}^{-1}X_{1,k/k-1}^{*}Z_{1,k/k-1}^{*T}\right]\ldots \left[W_{m}^{c}Z_{m,k/k-1}^{*}X_{m,k/k-1}^{*T}{\left(X_{m,k/k-1}^{*T}\right)}^{-1}{\left(X_{m,k/k-1}^{*}\right)}^{-1}X_{m,k/k-1}^{*}Z_{m,k/k-1}^{*T}\right]\\ \;-{\hat{z}}_{k/k-1}{\hat{x}}_{k/k-1}^{T}{\left({\hat{x}}_{k/k-1}^{T}\right)}^{-1}{\left({\hat{x}}_{k/k-1}\right)}^{-1}{\hat{x}}_{k/k-1}{\hat{z}}_{k/k-1}^{T}\\ =\sum_{i=1}^{m}W_{1}^{c}Z_{i,k/k-1}^{*}Z_{i,k/k-1}^{*T}-{\hat{z}}_{k/k-1}{\hat{z}}_{k/k-1}^{T} \end{array}$$

While

$$P_{zz,k/k-1}=\sum_{i=1}^{m}W_{1}^{c}Z_{i,k/k-1}^{*}Z_{i,k/k-1}^{*T}-{\hat{z}}_{k/k-1}{\hat{z}}_{k/k-1}^{T}+R_{k}$$

then,

(A4) $$P_{zz,k/k-1}-P_{xz,k/k-1}^{T}P_{k/k-1}^{-1}P_{xz,k/k-1}=R_{k}$$

Substitute Equation (A4) into Equation (A3), and due to $P_{k}^{-1}=Y_{k}^{}$ and $P_{k/k-1}^{-1}=Y_{k/k-1}^{}$, it can be get:

$$Y_{k}^{}=Y_{k/k-1}^{}+\left(Y_{k/k-1}^{}P_{xz,k/k-1}\right)R_{k}^{-1}{\left(Y_{k/k-1}^{}P_{xz,k/k-1}\right)}^{T}$$

□

## Appendix B

B. Verify that the update variance of the information vector at k+1 time is:

(A5) $${\hat{y}}_{k}=y_{k/k-1}+i_{k}$$

where $i_{k}=\left(Y_{k/k-1}P_{xz,k/k-1}\right)R_{k-1}^{-1}\left(v_{k}+P_{xz,k/k-1}^{T}{\hat{y}}_{k/k-1}\right)$.

**Proof.** The approximate estimate form of the gain matrix $K_{k}$ based on the linear minimum variance is:

(A6) $$K_{k}=P_{xz,k/k-1}P_{zz,k/k-1}^{-1}=Y_{k/k-1}^{-1}P_{yz,k/k-1}P_{zz,k/k-1}^{-1}$$

Meanwhile the state vector estimate is:

(A7) $$\begin{array}{ll} {\hat{x}}_{k} & ={\hat{x}}_{k/k-1}+K_{k}\left(Z_{k}-{\hat{z}}_{k/k-1}\right)\\ & ={\hat{x}}_{k/k-1}+Y_{k/k-1}^{-1}P_{yz,k/k-1}P_{zz,k/k-1}^{-1}\left(Z_{k}-{\hat{z}}_{k/k-1}\right) \end{array}$$

Multiply $Y_{k/k-1}^{}$ on both sides of Equation (A2), namely

(A8) $$Y_{k/k-1}^{}{\hat{x}}_{k}=Y_{k/k-1}^{}{\hat{x}}_{k/k-1}+P_{yz,k/k-1}P_{zz,k/k-1}^{-1}\left(Z_{k}-{\hat{z}}_{k/k-1}\right)$$

Substitute Equation (A1) into Equation (A8), it can be obtained:

$$\left(Y_{k}^{}-I_{k}^{}\right){\hat{x}}_{k}=Y_{k/k-1}^{}{\hat{x}}_{k/k-1}+P_{yz,k/k-1}P_{zz,k/k-1}^{-1}\left(Z_{k}-{\hat{z}}_{k/k-1}\right)$$

Due to $Y_{k/k-1}^{}{\hat{x}}_{k/k-1}={\hat{y}}_{k/k-1}^{}$ and $Y_{k}^{}{\hat{x}}_{k}={\hat{y}}_{k}^{}$, then

$$\begin{array}{ll} {\hat{y}}_{k}^{} & ={\hat{y}}_{k/k-1}+I_{k}^{}{\hat{x}}_{k}+P_{yz,k/k-1}P_{zz,k/k-1}^{-1}\left(Z_{k}-{\hat{z}}_{k/k-1}\right)\\ & ={\hat{y}}_{k/k-1}+\left(Y_{k/k-1}^{}P_{xz,k/k-1}\right)R_{k}^{-1}{\left(Y_{k/k-1}^{}P_{xz,k/k-1}\right)}^{T}{\hat{x}}_{k}+P_{yz,k/k-1}P_{zz,k/k-1}^{-1}\left(Z_{k}-{\hat{z}}_{k/k-1}\right)\\ & ={\hat{y}}_{k/k-1}+\left(Y_{k/k-1}^{}P_{xz,k/k-1}\right)R_{k}^{-1}{\left(Y_{k/k-1}^{}P_{xz,k/k-1}\right)}^{T}{\hat{x}}_{k}+\left(Y_{k/k-1}^{}P_{xz,k/k-1}\right)P_{zz,k/k-1}^{-1}\left(Z_{k}-{\hat{z}}_{k/k-1}\right)\\ & ={\hat{y}}_{k/k-1}+\left(Y_{k/k-1}^{}P_{xz,k/k-1}\right)R_{k}^{-1}\left[{\left(Y_{k/k-1}^{}P_{xz,k/k-1}\right)}^{T}{\hat{x}}_{k}+R_{k}^{}P_{zz,k/k-1}^{-1}\left(Z_{k}-{\hat{z}}_{k/k-1}\right)\right]\\ & ={\hat{y}}_{k/k-1}+\left(Y_{k/k-1}^{}P_{xz,k/k-1}\right)R_{k}^{-1}\left[{\left(Y_{k/k-1}^{}P_{xz,k/k-1}\right)}^{T}\left({\hat{x}}_{k/k-1}+Y_{k/k-1}^{-1}P_{yz,k/k-1}P_{zz,k/k-1}^{-1}\left(Z_{k}-{\hat{z}}_{k/k-1}\right)\right)+R_{k}^{}P_{zz,k/k-1}^{-1}\left(Z_{k}-{\hat{z}}_{k/k-1}\right)\right]\\ & ={\hat{y}}_{k/k-1}+\left(Y_{k/k-1}^{}P_{xz,k/k-1}\right)R_{k}^{-1}\left\{{\left(Y_{k/k-1}^{}P_{xz,k/k-1}\right)}^{T}{\hat{x}}_{k/k-1}+\left[{\left(Y_{k/k-1}^{}P_{xz,k/k-1}\right)}^{T}Y_{k/k-1}^{-1}Y_{k/k-1}^{}P_{xz,k/k-1}+R_{k}^{}\right]P_{zz,k/k-1}^{-1}\left(Z_{k}-{\hat{z}}_{k/k-1}\right)\right\}\\ & ={\hat{y}}_{k/k-1}+\left(Y_{k/k-1}^{}P_{xz,k/k-1}\right)R_{k}^{-1}\left[P_{xz,k/k-1}^{T}{\hat{y}}_{k/k-1}+\left[P_{xz,k/k-1}^{T}P_{k/k-1}^{-1}P_{xz,k/k-1}+R_{k}^{}\right]P_{zz,k/k-1}^{-1}\left(Z_{k}-{\hat{z}}_{k/k-1}\right)\right] \end{array}$$

According to Equation (A8), further simplification can be obtained:

$${\hat{y}}_{k}^{}={\hat{y}}_{k/k-1}+\left(Y_{k/k-1}^{}P_{xz,k/k-1}\right)R_{k}^{-1}\left[P_{xz,k/k-1}^{T}{\hat{y}}_{k/k-1}+v_{k}\right]$$

□
